# Glycosylation Is a Major Regulator of Phenylpropanoid Availability and Biological Activity in Plants

**DOI:** 10.3389/fpls.2016.00735

**Published:** 2016-05-26

**Authors:** Julien Le Roy, Brigitte Huss, Anne Creach, Simon Hawkins, Godfrey Neutelings

**Affiliations:** Centre National de la Recherche Scientifique, UMR 8576-Unité de Glycobiologie Structurale et Fonctionnelle, Université de LilleLille, France

**Keywords:** phenylpropanoids, glycosylation, UDP-glycosyltransferase, beta-glucosidase, lignin, flavonoids, compartmentalization

## Abstract

The phenylpropanoid pathway in plants is responsible for the biosynthesis of a huge amount of secondary metabolites derived from phenylalanine and tyrosine. Both flavonoids and lignins are synthesized at the end of this very diverse metabolic pathway, as well as many intermediate molecules whose precise biological functions remain largely unknown. The diversity of these molecules can be further increased under the action of UDP-glycosyltransferases (UGTs) leading to the production of glycosylated hydroxycinnamates and related aldehydes, alcohols and esters. Glycosylation can change phenylpropanoid solubility, stability and toxic potential, as well as influencing compartmentalization and biological activity. (De)-glycosylation therefore represents an extremely important regulation point in phenylpropanoid homeostasis. In this article we review recent knowledge on the enzymes involved in regulating phenylpropanoid glycosylation status and availability in different subcellular compartments. We also examine the potential link between monolignol glycosylation and lignification by exploring co-expression of lignin biosynthesis genes and phenolic (de)glycosylation genes. Of the different biological roles linked with their particular chemical properties, phenylpropanoids are often correlated with the plant's stress management strategies that are also regulated by glycosylation. UGTs can for instance influence the resistance of plants during infection by microorganisms and be involved in the mechanisms related to environmental changes. The impact of flavonoid glycosylation on the color of flowers, leaves, seeds and fruits will also be discussed. Altogether this paper underlies the fact that glycosylation and deglycosylation are powerful mechanisms allowing plants to regulate phenylpropanoid localisation, availability and biological activity.

## Introduction

Terrestrialization was probably one of the most important evolutionary steps in the history of life. After the transition from water to land, a part of the green lineage was anchored in the soil and became sessile. Unable to move, plants are continuously subjected to UV radiations, large temperature variations and other extreme growing conditions currently accentuated by climate change such as drought, waterlogging and cold stresses as well as increased ozone levels and other industrial pollutants. Furthermore, plants are attacked by a wide range of pathogens including viruses, fungi, nematodes and bacteria, and are also targeted by herbivores that use plants for food. Finally, since the development of agriculture, plants must increasingly deal with different types of pesticides. Both biotic and abiotic stresses represent unfavorable growth conditions negatively affecting plant growth and survival and leading to dramatic crop losses. On the other hand, plants also depend on abiotic and biotic agents for pollen and seed dispersal, on symbiotic microorganisms such as mycorrhizal fungi for water and mineral absorption, as well as rhizobia nitrogen-fixing bacteria in leguminous species. To manage both negative and positive interactions with the environment and other forms of life, individual plant species continually synthesize a complex and changing mixture of different secondary metabolites including phenylpropanoids, phenylpropanoid-derived compounds, terpenoids and alkaloids to respond to specific environmental situations (Timell, 1986; Cheynier et al., 2013; Mierziak et al., 2014; Pusztahelyi et al., 2015; Holbein et al., 2016; Ishihara et al., 2016). Although, a recent estimate put the number of secondary metabolites at more than 100,000 (Gachon et al., 2005) across plant species, it is likely that the real number is far higher. The increasing interest for metabolomics during the past decade has accelerated improvements in the sensitivity and specificity of spectrometric- and chromatographic-based technics as well as nuclear magnetic resonance (NMR) approaches.

**Figure 1:**
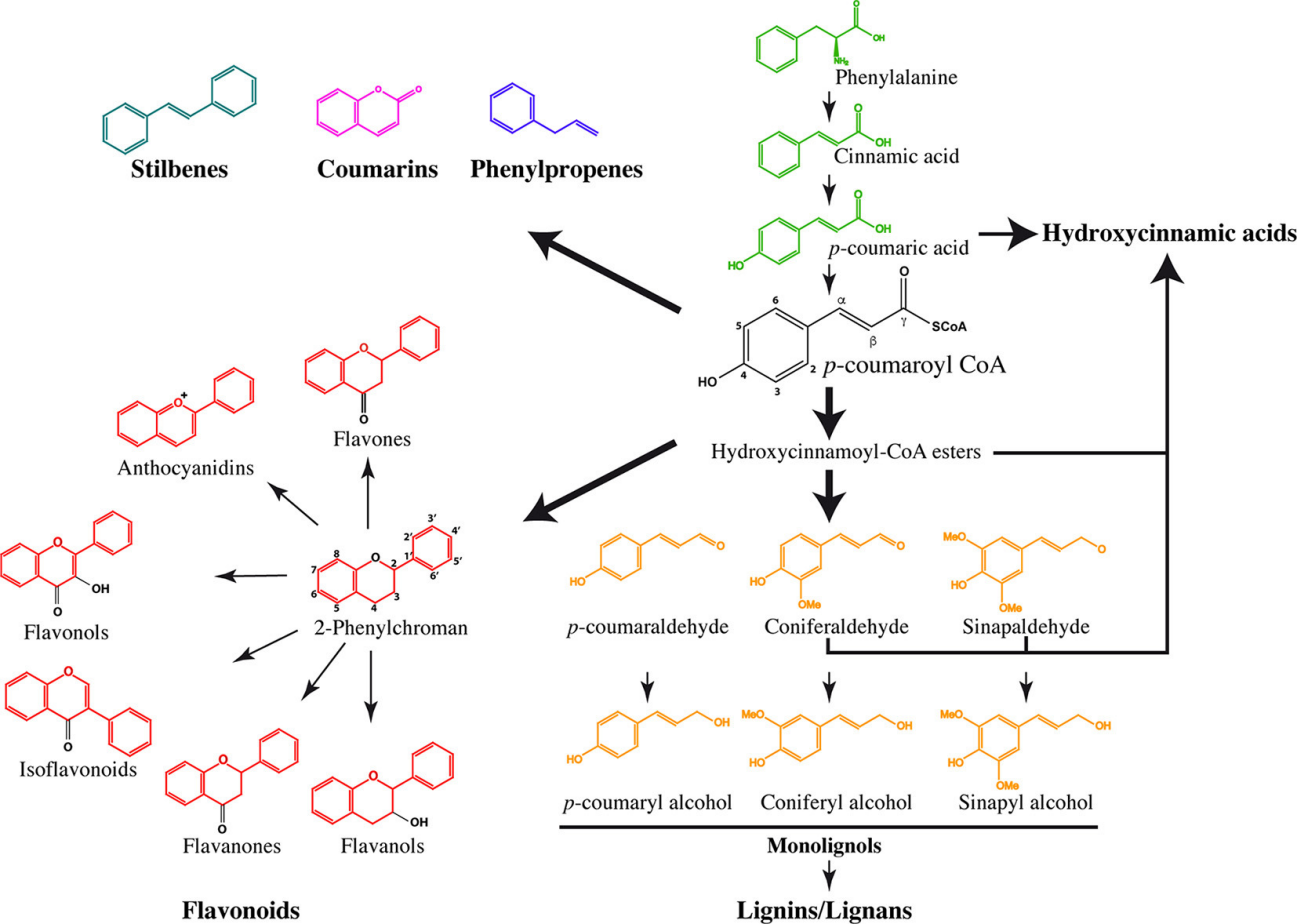
**Schematic view of the phenylpropanoid biosynthesis**. The general phenylpropanoid pathway begins by successive reactions resulting in the transformation of phenylalanine into *p*-coumaryl CoA, which is the common precursor of stilbenes, coumarins, phenylpropenes, 2-phenylchroman-containing flavonoids, and monolignols. The pathways leading to the production of hydroxycinnamic acids were obtained based on phenylpropanoid analysis in *Arabidopsis* mutants.

Phenylpropanoids are a large group of secondary metabolites containing a phenyl group linked to a 3-C propane side chain. Both the position of the propenyl double bond and the substituents on the benzene ring result in different compounds with diverse biological activities (Koeduka et al., 2014). In many different species, the genes involved in the phenylpropanoid pathway belong to multigenic families (Ehlting et al., 1999) and are mainly regulated by MYB and NAC transcription factors (Sablowski et al., 1994; Zhong et al., 2006). The first enzymatic step is catalyzed by phenylalanine ammonia lyase (PAL), which converts phenylalanine issued from the shikimate pathway into cinnamic acid (Figure 1), which in turn is transformed to *p*-coumaric acid by cinnamate 4-hydrolase (C4H). Hydroxycinnamates (HCAs) derived from *p*-coumaric acid, CoA esters or hydroxycinnamaldehydes constitute the simplest phenylpropanoids (Strack, 2001). They can accumulate as ester- or amide-conjugates with monosaccharides, organic acids, lipids and amines and, when activated with CoenzymeA (CoA) or glucose, become acyl donors for modifications of secondary metabolites. HCAs are involved in the plant's response to pathogens and interactions with symbionts (Babst et al., 2014). Grass cell walls are characterized by large amounts of HCAs such as ferulic and *p*-coumaric acids. In these species, ferulic acid binds to lignin, polysaccharides and structural proteins and leads to their cross-linking but they are also present in smaller quantities in the cell walls of most dicots (for review see de Oliveira et al., 2015). In sugarcane, sinapic, *p*-coumaric and ferulic acids are deposited during the early stages of lignification, and levels of hydroxycinnamic acids were higher in parenchyma walls than in the vascular bundles (He and Terashima, 1990, 1991). They can also be integrated into lignins being involved in ligno-polysaccharide cross-links (Tobimatsu et al., 2012). The subsequent production of *p*-coumaroyl CoA by 4-coumaroyl CoA-ligase (4CL) constitutes an important branch point (Ehlting et al., 1999) leading to the biosynthesis of the different subfamilies of phenylpropanoid-derived molecules (Vogt, 2010), the most important being undoubtedly lignin, the second most abundant plant polymer on the Earth's surface.

Lignin heteropolymers are derived mainly from three different monolignols: *p*-coumaryl alcohol, coniferyl alcohol, and sinapyl alcohol, synthesized in the cytoplasm through at least 10 different enzymatic reactions and are oxidized by laccases and/or peroxidases prior to polymerization into the lignin polymer (Freudenberg, 1968; Sterjiades et al., 1992; Boerjan et al., 2003). Instead of being exported to the apoplast, monolignols can also be stored as glycosylated conjugates in the vacuole (Dima et al., 2015). Lignins play a crucial role during the plant's life by providing cell integrity, mechanical support, water transport, assimilation through the vascular system and are also involved in defense mechanisms. (Neo)lignans are found in a wide range of species and represent a large group of diverse phenolics derived from the oxidative dimerization of two monolignols. Lignans are dimerized on their C3 side chains in a tail to tail structure whereas neolignans result from a head to tail dimerization (Cheynier et al., 2013). Flaxseed lignans have gained particular interest since some of them (e.g. SDG: secoisolariciresinol diglucoside) have potential antimutagenic, anti-inflammatory, anti-oxydant, antimicrobial and antiobesity effects (Imran et al., 2015).

Flavonoids are ubiquitously present across the plant kingdom. They are synthesized from the condensation of *p*-coumaroyl-CoA with three malonyl-CoA molecules by a chalcone synthase (CHS), which in turn produces a flavanone containing a 2-phenylchroman backbone. The same 2-phenylchroman backbone also gives rise to flavanols, isoflavonoids, flavonols, flavones and anthocyanidins. These compounds do not have a typical phenylpropanoid structure and are therefore considered as phenylpropanoid-derived compounds (Stafford, 1991). The different carbons of the flavonoid skeleton show numerous substitutions catalyzed by isomerases, reductases, hydroxylases, methylases, and glycosyltransferases leading to high structural diversity (Harborne and Williams, 2001; Ferrer et al., 2008).

Coumarins play roles in plant defense against fungi and possess both antioxidative and antimicrobial activities (Kai et al., 2006) as they can scavenge reactive oxygen intermediates produced during the hypersensitive response (Chong et al., 2002). The core structure derives from cinnamates via *ortho*-hydroxylation of the aromatic ring, *trans*/*cis* isomerization and lactonization. They are widely distributed in the plant kingdom and are classified into several categories depending on whether they harbor simple hydroxyl, alkoxy and/or alkyl groups in the benzene ring, or if additional benzene rings are present (Shimizu, 2014). Only 12 plant families have been listed to synthesize stilbenes from flavonoids precursors through stilbene synthases (Holl et al., 2013). There is a particular interest for *trans*-resveratrol synthesized in grapevine, cranberry, blueberry and peanut because of its pharmacological properties associated with protection toward cardiovascular, neurodegenerative diseases, cancer, and diabetes. Finally, phenylpropenes are volatiles emitted to attract both pollinators—through fragrances and seed dispersers through fruit scents. When they are produced in vegetative tissues, they also act as deterrents, toxicants and antifeedants to repel herbivores and bacterial pathogens.

The basic phenylpropanoid molecular backbone is synthesized in one of the numerous core metabolic pathways invented by plants during evolution, but the massive expansion of these different subfamilies of molecules is mainly due to the grafting of side chains on the basal structures. Among these modifications, glycosylation appears as a major reaction leading not only to an increase in structural diversity but also to an increase in the diversity of their properties. The glycosylation status of phenylpropanoids is regulated by the combination of regioselective glycosyltransferases (GTs) and glycoside hydrolases (GHs) with relatively broad substrate spectrum. Glycosyltransferases catalyze the transfer of sugar moieties from activated donor molecules to specific acceptor molecules such as sugars, lipids, proteins, or small molecules including phenylpropanoids. Information on GHs and GTs, as well as polysaccharide lyases and carbohydrate esterases, can be found in the CarbohydrateActive enZymes (CAZy) database (Davies et al., 2005) and a specific PlantCAZyme database is now also available (Ekstrom et al., 2014).

CAZy takes into account similarities in amino acid sequences, 3D-structures, sugar donors, transferred sugars, acceptors, catalytic mechanisms (inverting or retaining mechanisms) and more recently the combination of modules which can be catalytic or not (Lombard et al., 2014). Among the 98 GT families present in the CAZy database in may 2016, the GTs catalyzing secondary metabolite conjugation all belong to the GT1 family and are commonly named UDP-glycosyltransferases (UGTs) as UDP-sugars are used as the sugar donor. When they act on the conjugation of secondary metabolites, most UGTs perform O-glycosylation, but on some xenobiotics, UDP-glucose can act as sugar donor for S-, C- or N-glycosylations. Pollutants such as 2,4,5-trichlorophenyl and 3,4-dichloroaniline can potentially be metabolized by 44 different UGTs in *Arabidopsis* through O- and N-glucosylation, respectively, whereas one UGT (UGT72B1) was shown to act as a bifunctional enzyme carrying both activities (Brazier-Hicks et al., 2007). Conjugated xenobiotics may then be exported from the cell to the apoplast or imported into the vacuole. C-glucosylation of 2-hydroxyflavanone precursors of flavonoids was also reported for the rice OsCGT (Brazier-Hicks et al., 2009). S-glycosylation takes place during the biosynthesis of glucosinolates involved in plant defense against pathogen and herbivores (Grubb et al., 2004).

When an acceptor presents multiple possible binding sites for a sugar, UGTs exhibit regioselectivity by transferring the sugar to a specific position. This was demonstrated mainly for different coumarins (Lim et al., 2003) and flavonoids for which prediction of regioselectivity was assessed (Jackson et al., 2011). The glycosylation status is also regulated by glycoside hydrolases (GHs) involved in hydrolysis and/or rearrangement of glycosidic bonds. Among these, several β-glucosidase subfamilies can remove the non-reducing terminal ß-D-glucosyl from glucoconjugates. Most β-glucosidases are included in the CAZy GH1 family (Rouyi et al., 2014) but others can be found in GH3, GH5, GH9, GH30, and GH116 families. The different GH families contain enzymes showing different substrate specificities (Lombard et al., 2014) and most likely broad range specificities because of the high number of glycoconjugates within the plant compared to the number of ß-glucosidases (Ketudat Cairns et al., 2015).

In this present review, we will first summarize the current knowledge on UGTs and β-glucosidases related to the glycosylation status of phenylpropanoids and then we will evaluate the impact of sugar conjugation on their compartmentation. The function of these glucoconjugates will also be discussed in the context of lignification, development and the stress response.

## Catalytic activities regulating phenylpropanoid glycosylation

### UDP-glycosyltransferases

**Figure 2:**
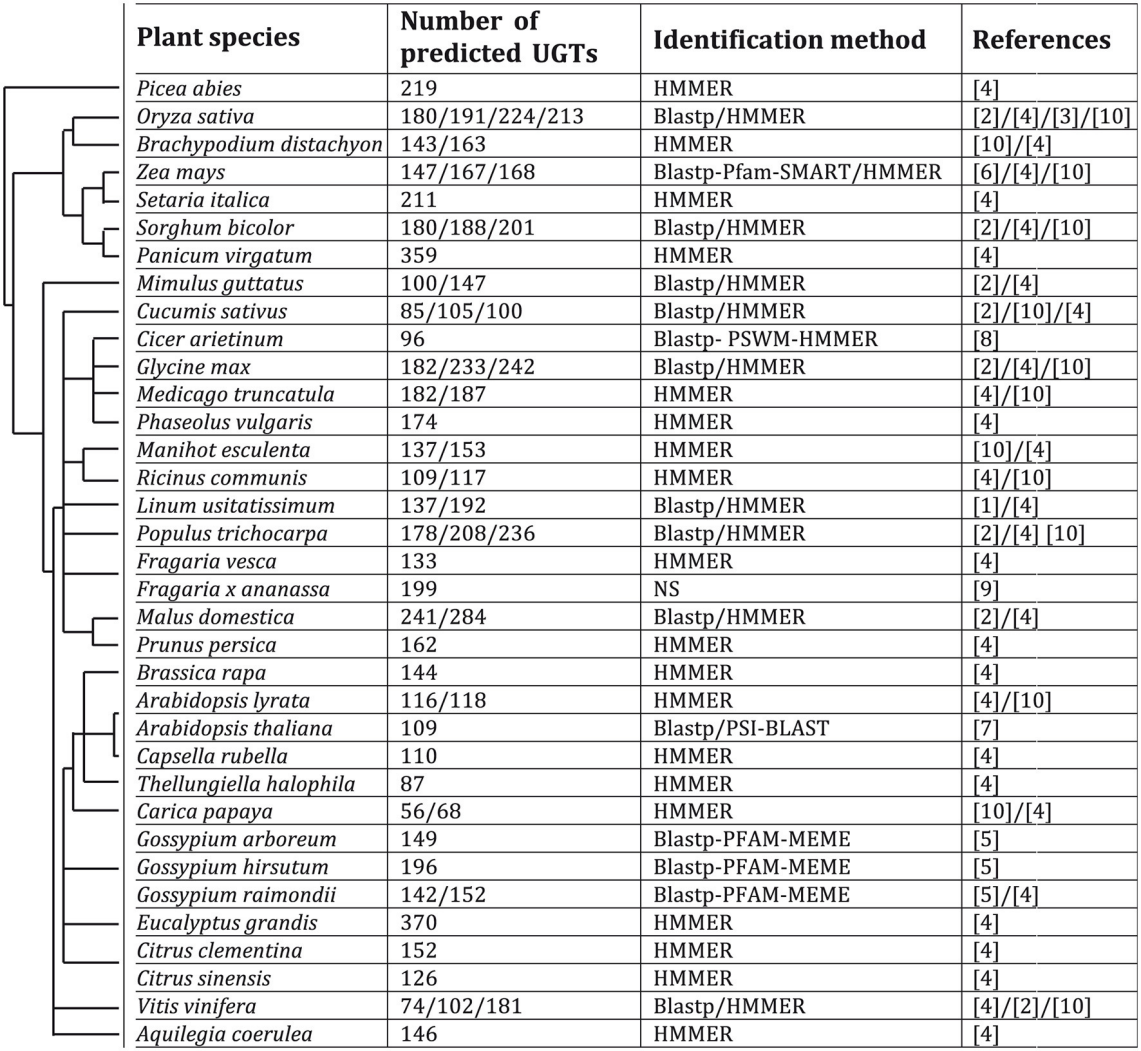
**Predicted UGT enzymes in different higher plants**. Species are arranged in an order based on taxonomic families. [1], Barvkar et al. (2012); [2], Caputi et al. (2012); [3], Cao et al. (2008); [4], Ekstrom et al. (2014); [5], Huang et al. (2015); [6], Li et al. (2014); [7], Ross et al. (2001); [8], Sharma et al. (2014); [9], Song et al. (2015); [10], Yonekura-Sakakibara and Hanada (2011).

UGTs are usually named with a specific nomenclature including family numbers 1–50 used for animals, 51–70 for yeasts, 71–100 for plants and 101–200 for bacteria followed by a letter for the sub-family defined by proteins sharing at least 60% homology, and finally an arabic number to describe the gene (Mackenzie et al., 1997; Ross et al., 2001). According to the nomenclature committee of the international union of biochemistry and molecular biology (IUBMB), they are also named as EC 2.4.1.x, EC 2.4.2.x, and 2.4.99.x. UGTs include enzymes able to transfer a sugar linked to uridine diphosphate (UDP) to a large range of acceptors. In plants, the nucleotidic sugar donor is mostly glucose but can also be galactose, xylose, rhamnose, arabinose, or glucuronic acid, the latter also being the main sugar derivative donor for UGTs in mammalians (Ross et al., 2001; Osmani et al., 2008; Yonekura-Sakakibara et al., 2008). The conjugation of acceptor molecules such as hormones, xenobiotics or secondary metabolites by UGTs allows the plant cell to modulate their biochemical proprieties and thus has a strong influence on their biological activity and compartment storage. Although, the primary sequences of the UGT proteins show only low similarities (Vogt and Jones, 2000), a highly conserved 44-amino acid sequence named plant secondary product glycosyltransferase (PSPG)-box believed to be involved in binding to the UDP moiety, is present in the C-terminal region (Mackenzie et al., 1997). This sequence is therefore commonly used as a query to identify the UGT genes in plant genomes by using the Blastp program in addition to position-specific weight matrix (PSWM) guided search and a hidden Markov model (HMMER) program (Figure 2). The sequences can then be confirmed by using the MEME suite or the Pfam (http://pfam.sanger.ac.uk/search), SMART (http://smart.embl-heidelberg.de/) and Interpro (https://www.ebi.ac.uk/interpro/) databases. The very large spectrum of molecules potentially glycosylated by UGTs is in accordance with the high number of sequences identified at a genome-wide scale in plants as compared to other kingdoms including animals. However, there does not seem to be any relationship between the number of predicted UGT models and the taxonomic families such as liliopsida and eudicotyledons.

Among the numerous phenylpropanoids, flavonoids are probably among the best-characterized molecules in terms of glycosylation due to their numerous medical and commercial benefits. The basic structure of flavonoids consists of two phenyl groups joined by three carbons cyclized with oxygen (Figure 1). On this double ring, the carbons are annotated from 2 to 8 and on the third ring anchored on C2 they are annotated from 1′ to 6′. Successive glycosylation by UGTs is possible on ring positions bearing hydroxyl groups mostly on C3 and C7. In *Arabidopsis*, three homologous enzymes belonging to the UGT78D family catalyze the first 3-*O*-glycosylation steps on flavonols. These three enzymes share the same aglycone substrates kaempferol, quercetin and isorhamnetin but differ by their specificity toward the donor sugar, with UGT78D1 using UDP-rhamnose, UGT78D2 using UDP-glucose and UGT78D3 using UDP-arabinose (Yonekura-Sakakibara et al., 2008). The following step consists of a 7-*O*-glycosylation during which rhamnose is transferred by UGT89C1 or glucose by UGT73C6. Whether this transfer takes place or not, a secondary glycosylation step is also possible on the conjugated C3 by addition of a glucose on the flavonoid 3-O-glucoside by UGT79B6 (Yonekura-Sakakibara et al., 2014), or a rhamnose via a 1′-2″ link forming the triglycoside kaempferol 3-O-beta-[alpha-rhamnopyranosyl(1–>2)-glucopyranoside]-7-O-alpha-rhamnopyranoside. In the same metabolic pathway, recombinant protein or mutagenesis approaches showed that the production of anthocyanins also depended on the transfer of sugar moieties onto the carbon rings. Both UGT78D2 and UGT75C1 were identified in *Arabidopsis* as glucosyltransferases specific to the C3 and C5 positions, respectively (Tohge et al., 2005; Kubo et al., 2007) and UGT79B1 was shown to be involved in anthocyanin biosynthesis by converting cyanidin 3-*O*-glucoside to cyanidin 3-*O*-xylosyl(1→2)glucoside (Yonekura-Sakakibara et al., 2012). To a lesser extent the same enzyme is also capable of recognizing cyanidin 3-*O*-rhamnosyl (1→2) glucoside as substrate. Beyond the *Arabidopsis* model, numerous different UGT activities on flavonoids were identified in medicinal and aromatic plants: a flavonoid 7-*O*-glucuronosyltransferase in *Perilla frutescens* and *Scutellaria laeteviolacea* (Noguchi et al., 2009), a flavonoid glucoside: 6″-*O*-glycosyltransferase in *Catharanthus roseus* (Masada et al., 2009) or a flavonol 3-*O*-glucoside: 2″-*O*-glucosyltransferase in *Crocus sativus* (Trapero et al., 2012). The identification of the complete plant set of flavonoid-associated UGTs is still ongoing and major advances will undoubtedly be made with the growing number of full genome sequences available.

The identification and characterization of UGTs used to construct final complex molecular structures such as flavonoids or anthocyanins can be quite straightforward but is less obvious for those acting on more upstream intermediates such as hydroxycinnamic acids, aldehydes or alcohols. The role of these UGTs may indeed be difficult to reveal because of the proximity of related UGT genes in the *Arabidopsis* genome preventing generation of multiple mutants (Meißner et al., 2008). The use of techniques allowing fine gene targeting such as CRISPR-Cas9 may allow circumventing this constraint in the future. In addition the presence of overlapping substrates between several enzymes and the broad range of substrates recognized under *in vitro* conditions by each enzyme may also be a source of difficulty. One example is the TOGT1 recombinant enzyme derived from a tobacco gene and active toward ferulic, caffeic, cinnamic, *p*-coumaric, and *o*-coumaric acids as well as coniferyl alcohol (Fraissinet-Tachet et al., 1998) although it is important to be cautious with respect to the results obtained by *in vitro* activity approaches. In *Arabidopsis*, the UGT72E subfamily was extensively studied by the group of D. Bowles in the context of their possible implication in regulating lignin precursor availability (Lim et al., 2005). A series of substrates was tested on the recombinant proteins and results showed that UGT72E1 and UGT72E2 displayed catalytic activities on coniferyl and sinapyl aldehydes and that the monolignols coniferyl and sinapyl alcohols were glycosylated by UGT72E2 and UGT72E3 to the respective 4-O-ß -D-glucosides forms (coniferin and syringin; Lim et al., 2001, 2005). It should be noted that the enzymatic tests were performed on HCAs and on most of the intermediates of the lignification biosynthetic pathway but in general the CoA esters were omitted most likely because they have only recently become commercially available.

When used as a substrate by glucosyltransferases, caffeic acid can form caffeoyl-3-*O*-glucoside and caffeoyl-4-*O*-glucoside but also 1-*O*-caffeoylglucose, a high-energy glucose ester potentially used as a donor molecule during the formation of various hydroxycinnamic acid *O*-esters in plants (Mock and Strack, 1993; Lim et al., 2003). In the same way, 1-*O*-sinapoylglucose is formed by an UDP-glucose:sinapate glucosyltransferase (SGT) enzymatic activity and serves as a sinapoyl donor in various acyltransfer reactions leading to the accumulation of sinapate esters (Milkowski and Strack, 2010). During seed development in *Brassicaceae*, it is converted to sinapoylcholine (sinapine), the major seed phenylpropanoid that accumulates to levels of 1–2% of seed dry weight (Baumert et al., 2005), and is then further hydrolyzed in germinating seeds (Milkowski and Strack, 2010). In the seedling, sinapoylglucose is also channeled toward sinapoylmalate (2-*O*-sinapoyl-L-malate), a UV-B sunscreen protectant that accumulates in the vacuoles of the leaf epidermis and causes the adaxial surface to fluoresce in blue under UV light (Sinlapadech et al., 2007). In *Arabidopsis*, recombinant proteins of the four members of the UGT84A clade can convert HCA to 1-*O*-ß-glucose esters (Milkowski et al., 2000) and the overexpression of *UGT84A1, UGT84A2*, and *UGT84A3* leads to an increased level of sinapoylglucose in seeds and seedlings (Meißner et al., 2008). In *Brassica napus*, the Sinapoylglycosyltransferase (SGT) activity is due to UGT84A9, an enzyme with glucosylating capacities on sinapate, cinnamate, ferulate, 4-coumarate, and caffeate (Milkowski et al., 2000). The closely related UGT84A10 can also catalyze the formation of HCA glucose esters with preference for ferulate and sinapate when produced in *E. coli* (Mittasch et al., 2007). Recent work showed that the suppression of *UGT84A9* in RNAi lines induced strong metabolic modifications in late stages of seed development confirming the essential upstream role of the SGT activity on metabolites whose synthesis depends on 1-*O*-sinapoylglucose (Hettwer et al., 2016). A similar SGT activity in *Populus* has been assigned to GT1-316. Interestingly, the corresponding gene has a higher expression in plants grown under N-limiting conditions. Similarly, a stress response of the *Fragaria* × *ananassa FaGT2* gene was also observed after oxidative stress treatments in both strawberry fruit and cell cultures confirming that the final acceptors probably play an important role in the stress resistance mechanisms (Lunkenbein et al., 2006).

**Figure 3:**
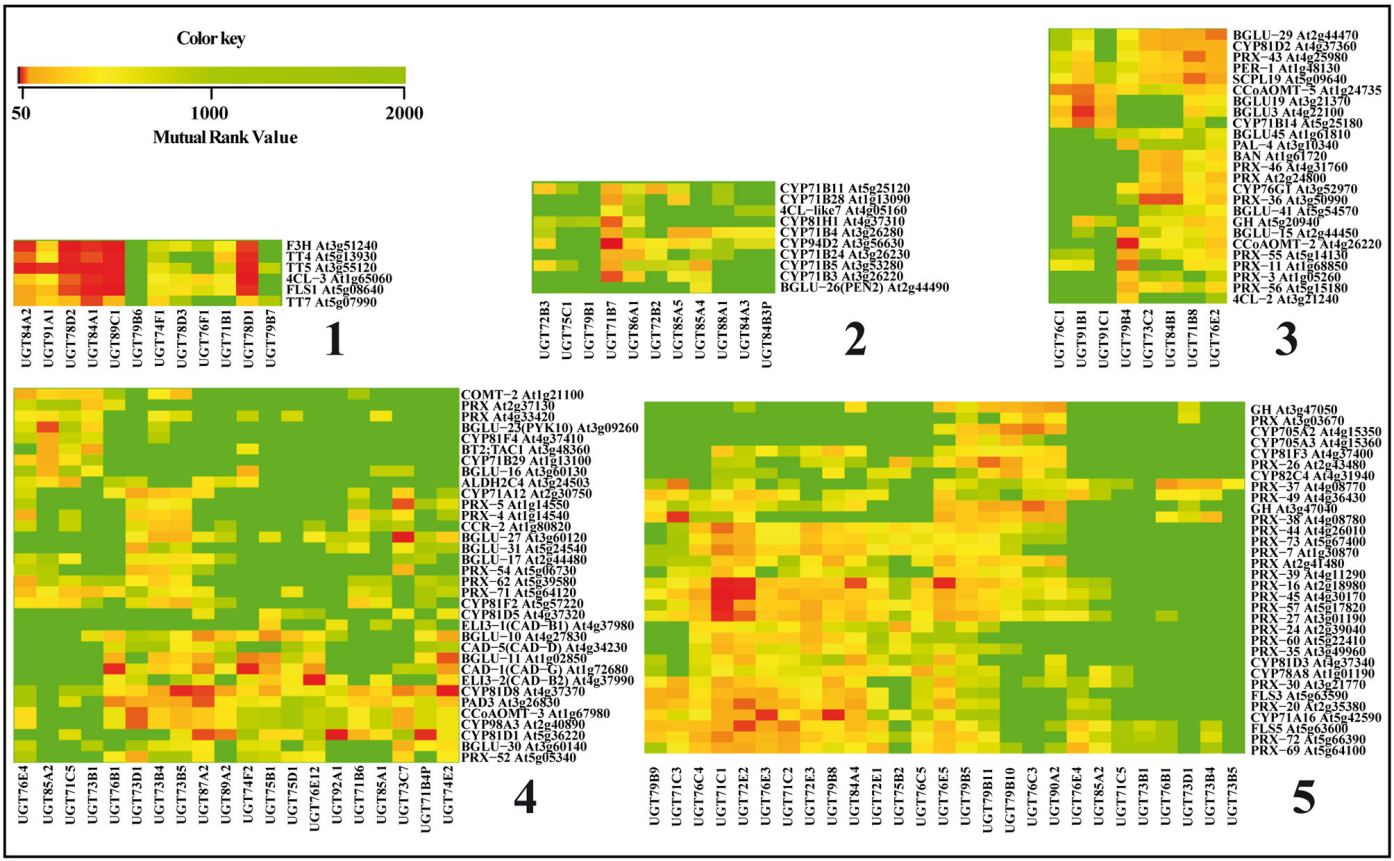
**Co-expression of UGT and phenylpropanoid genes**. Five relevant groups of coexpressed genes delimited by hierarchical clustering indicated on the left of the heatmap were extracted from the global heat map presented in Supplementary Figure 1. Groups 1, 2, and 5 contain genes known to be involved in the glycosylation of the major phenylpropanoids and groups 3 and 4 contain genes with high coregulation values. When the mutual rank (MR) value <0, there is co-expression a50, co-expression between the genes is considered as strong; if 51 < *MR* < 1000, there is co-expression and if MR>1001, there is no co-expression.

To reveal potential relations between genes involved in the phenylpropanoid backbone construction and those used by the plant for regulating the glycosylation status, we performed a mutual ranking classification using ATTED-II (Obayashi et al., 2009). This coexpression database (v8.0; March 2016) contains 58 experiments, 1388 GeneChips collected by AtGenExpress and about 20,000 publicly available files to provide gene-to-gene mutual ranks. We focused on a specific comparison between the known phenylpropanoid synthesis genes and the UGT genes found to be expressed in *Arabidopsis*. The complete results are presented as a heat map in Supplementary Figure 1 and a focus on five zones determined by hierarchical clustering containing a high number of coregulated genes is presented in Figure 3. The UGT genes known to be involved in the glycosylation of the major phenylpropanoids are included in groups 1, 2, and 5. When the flavonoid biosynthesis pathway is considered (Figure 3, group 1), UGT78D1, UGT78D2, and UGT89C1 are all highly co-regulated with the four successive genes involved in the transformation of *p*-coumaroyl CoA to kaempferol/quercetin i.e., chalcone synthase (TT4), chalcone isomerase (TT5), flavanone 3-hydroxylase (F3H) and flavonol synthase (FLS). Only the three latest genes are coexpressed with *UGT78D3*. In the same group, the sinapoylglucose specific genes *UGT84A1* and *UGT84A2* are coexpressed with the flavonoid pathway genes, which is in accordance with the fact that this component is also a substrate for anthocyanin sinapoyltransferase (Fraser et al., 2007). *UGT75C1* and *UGT79B1* are present in group 2 but are not coregulated with the corresponding phenylpropanoid genes.

### ß-glucosidases

ß-glucosidases (EC 3.2.1.21) have been described in Archaea, Eubacteria, and Eukaryotes (Ketudat Cairns and Esen, 2010). In plants, they show many different quaternary structures and can form dimers (Dharmawardhana et al., 1995), tetramers (Hösel et al., 1978) or decamers (Fan and Conn, 1985). In *Cassava*, linamarases can also be present as homooligomers with different numbers of monomers forming very large aggregates with sizes up to 2000 kDa (Sermsuvityawong et al., 1995). They catalyze the hydrolysis of terminal, non-reducing β-D-glucosyl residues with release of β-D-glucose and the corresponding free aglycone from various substrates including glucosides, 1-*O*-glucosyl esters and oligosaccharides. ß-D-glucosidase activity is among the oldest enzymatic activity known as it was reported in the nineteenth century that almond emulsin was able to cleave the cyanogenic glucoside amygdalin (Wöhler and Liebig, 1837).

Plant glycosylated phenylpropanoids are not just substrates for endogenous (plant) enzymes but are also potential targets for ß-glucosidases from many different organisms. In yeast, hydrolytic activity toward 7- or 4′-*O*-glucosides of isoflavones, flavonols, flavones, and flavanones can be attributed to 3 genes and is probably related to metabolism of flavonoids from plant material within the culture broth (Schmidt et al., 2011). During human digestion, monoglycosylated and aglycone forms of flavonoids are more easily absorbed than the original multi-glycosylated compound (Vila-Real et al., 2011). The absorption of dietary flavonoid glycosides includes a critical deglycosylation step which is thought to occur in the oral cavity by microbiota showing β-glucosidase activity necessary for the delivery of the biologically active aglycones to epithelial cell surfaces (Walle et al., 2005). The plants themselves also show ß-glucosidase activity toward a large range of their own phenylpropanoids underlying the fact that the glycosylated forms of these molecules are unlikely to represent dead-end products. Several examples of the implication of deglycosylated phenylpropanoids in defense have been clearly illustrated (König et al., 2014). Nevertheless, this enzymatic activity has also been proposed to mediate the turnover of flavonol biglycosides and may therefore have important consequences on plant growth and development (Roepke and Bozzo, 2015).

On a genome-wide scale, the number of ß-glucosidase genes has only clearly been determined in a small number of species. In *Arabidopsis*, 47 GH1 ß-glucosidase genes annotated from *BGLU1* to *BGLU47* were shown to have a potential common evolutionary origin according to their position within the phylogenetic tree (Xu et al., 2004). The first global analysis of the rice GH1 family identified at least 31 genes (of 34 apparently functional genes) expressed in different organs and growth stages (Opassiri et al., 2006). In this species, duplication events occurring after the divergence with the *Arabidopsis thaliana* lineage gave rise to most of the identified genes. Although, the global number of predicted gene models is higher in maize compared to *Arabidopsis*, only 26 ß-glucosidase paralogs were identified in the inbred line B73. Gomez-Anduro et al. (2011) explained this difference by the fact that a glucosinolate-mediated biotic stress response is absent in maize, reducing the amount of glycosylated substrates. The number of GH1 genes compared to the global number of gene models was also low in *Sorghum bicolor* (Sekhwal et al., 2013) suggesting that this feature may be characteristic of liliopsida genomes, however this should be confirmed by an in depth analysis of a much larger number of plant genomes based on the CAZy annotation. In agreement with the large number and diversity of glycoconjugates present in a given plant and the limited number of ß-glucosidases, these enzymes present overlapping specificities. For example, the recombinant BGLU45 and BGLU46 from *Arabidopsis* hydrolyze the glycosylated monolignols syringin, coniferin, and *p*-coumaryl alcohol 4-O-ß-D-glucoside (Escamilla-Trevino et al., 2006) but BGLU45 preferentially hydrolyses syringin whereas BGLU46 presents a broader specificity to other phenolic glucosides. This promiscuity is exploited during *in vitro* assays since many ß-glucosidases are first tested with *p*-nitrophenyl-sugars before a more in depth characterization of the preferred cleaved sugar (Escamilla-Trevino et al., 2006; Baiya et al., 2014). On the contrary, other ß-glucosidases seem to show a more strict specificity for their natural glycosylated compounds (Ketudat Cairns et al., 2015). For instance, the *Arabidopsis* BGLU21, BGLU22, and BGLU23 exclusively cleave the coumarin scopolin into the aglycone scopoletin but do not hydrolyse *p*- or *o*-nitrophenyl-sugars (Ahn et al., 2010).

During lignin biosynthesis, plant cells produce the monolignols *p*-coumaryl alcohol, coniferyl alcohol and sinapyl alcohol. Their corresponding glucoconjugates *p*-coumaryl alcohol glucoside, coniferin, and syringin can be used as substrates by a specific group of coniferin ß-glucosidases. These coniferin β-glucosidases (EC 3.2.1.126) belong to the GH3 family and were first described in *Picea abies* seedlings (Marcinowski and Grisebach, 1978) and in *Cicer arietinum* suspension cells (Hösel et al., 1978). In both species, the enzymes deglycosylated coniferin as well as syringin. It was previously speculated that the role of these specific enzymes could be to hydrolyse monolignol glucosides prior to incorporation of the aglycones in the lignin macromolecule by oxidative radical coupling thereby regulating the amount of precursors for lignification. Lignins from gymnosperms are rich in G units (Weng and Chapple, 2010) and support for a possible role of β-glucosidases in lignification was provided by the observation that high amounts of coniferin are accumulated in the cambium together with the co-localization of coniferin ß-glucosidase and peroxidase activity in the differentiating xylem of *Pinus contorta* (Dharmawardhana et al., 1995). The presence of both glucosylated monolignols and ß-glucosidase activity was also previously reported in *Pinus banksiana* (Leinhos and Savidge, 1993). Despite such observations, the use of radioactive precursors in *Pinus contorta* indicated that coniferin was probably not the main source of the coniferyl alcohol incorporated in lignins (Kaneda et al., 2008). The presence of coniferin ß-glucosidase is not exclusive to gymnosperms and these enzymes have also been found in vegetative and reproductive tissues of *Oryza sativa* (Baiya et al., 2014), in the xylem of *Betula pendula* (Marjamaa et al., 2003) and in the interfascicular fibers and protoxylem of *Arabidopsis* (Chapelle et al., 2012). The relation between lignification and monolignol 4-O-ß -D-glucosides is further discussed below.

## The role of glycosylation in the cellular compartmentation

One of the most predominant questions about phenylpropanoid biosynthesis concerns the spatial distribution of both precursors and final products within the cells. Many phenylpropanoid molecules necessary for plant development and/or defense are also toxic and an important challenge for plants is how to successfully manage the production/storage of these potentially dangerous molecules (Väisänen et al., 2015). While the association between PAL and C4H enzymes on microsomal membranes is believed to be responsible for the rapid transformation of phenylalanine to 4-coumaric acid in tobacco (Rasmussen and Dixon, 1999), much less evidence exists to support the existence of any large scale, tight metabolic channeling that could prevent accumulation of intermediates synthesized in the cytosol. The vacuole is one major compartment that has often been identified as an important site of storage for phenylpropanoid-derived products. Coniferin was shown to be accumulated in protoplasts of pine cambial cells, most likely in the vacuoles (Leinhos and Savidge, 1993) and recent phenolic profiling of vacuoles from *Arabidopsis* leaves identified coniferin, coniferin hexoside, and 34 other diverse glycosylated phenolics including derivatives of coniferyl alcohol, sinapyl alcohol, ferulic acid, and sinapic acid (Dima et al., 2015).

The cell wall is another major compartment where the oxidation machinery including peroxidases and laccases forms monolignol free-radicals that are subsequently cross-coupled in a non-stereospecific reaction to form lignin (Boerjan et al., 2003). In the apoplast, two monolignols can also undergo an enantioselective radical coupling reaction controlled by dirigent proteins to form lignans (Davin et al., 1997). Several lines of evidence support the biosynthesis of lignans in this compartment including the presence of the N-terminal signal peptide in dirigent proteins (Ralph et al., 2006), immunolocalization of these proteins in the cell wall (Burlat et al., 2001), and their presence in apoplastic protein extracts (Uzal et al., 2009). A previous report assuming that (neo)lignans were also located in vacuoles of *Linum* cell cultures (Kuhlmann et al., 2002) was confirmed recently by their detection in *Arabidopsis* leaf vacuoles (Dima et al., 2015). This paper also reported for the first time that in *Arabidopsis*, the combinatorial radical coupling reactions leading to (neo)lignans synthesis usually detected in the apoplast could also be detected in the cytoplasm. This observation greatly contributes to resolving the apparent paradox of how oligolignol hexosides arise in plant cells. Logically their formation requires an oxidative coupling reaction—usually thought to occur exclusively in the cell wall, followed by a glycosyl conjugation occurring in the cytoplasm. Nevertheless, this explanation does not totally exclude the possibility that a proportion of the cytoplasmic oligolignols may be formed in the apoplasm and subsequently transferred to the cytoplasm via specific (neo)lignin transporters as in mammalian systems (Miguel et al., 2014).

The glycosylation step is essential for the synthesis, transport, and sequestration of some phenylpropanoids. Because of their abundance and their role in flower coloration, the transport of glycosylated anthocyanins has been extensively studied over the past years (Yazaki, 2006). The involvement of Multidrug Resistance-associated Protein (MRP) also called ATP Binding Cassette subfamily C (ABCC) transporters in the vacuolar transport of such phenolic glucosides was first proposed in maize (Marrs et al., 1995) and later in carnation (Larsen et al., 2003) and *Arabidopsis* (Kitamura et al., 2004). However, these transporters are known to translocate a wide range of glutathione (GSH)-conjugated substrates through the membranes and anthocyanin-GSH conjugates have never been detected in plants (Ohkama-Ohtsu et al., 2011). Nevertheless, a potential co-transportation of GSH and glycosylated anthocyanidins was suggested by the heterologous expression of grapevine *ABCC1* in yeast (Francisco et al., 2013). A similar transportation system must still be validated in other species. In the lignification process, the glycosylation of hydroxycinnamic aldehydes and alcohols has been proposed to form part of an overall regulatory network of lignin precursor homoeostasis. In this model, ABC-like transporters located in the plasma and vacuolar membranes show differential affinities toward glycosylated and non-glycosylated molecules thereby controlling subcellular compartmentation (Miao and Liu, 2010). The 4-*O*-glycosylated forms are preferentially transported (in the presence of ATP) across the tonoplast into the vacuole whereas the specificity of the plasma membrane transporters is more relaxed with respect to ATP-dependence and favors the passage of aglycone forms. Given the existence of such preferential transport, it is tempting to speculate that unglycosylated lignin precursors are transported across the plasma membrane by specific ABC-like transporters and undergo free-radical polymerization while a surplus of monolignols could be glycosylated and transferred to the vacuole for storage by a transporter specific to glycoconjugates. *ABC* genes constitute large multigene families (approximately 130 members in *Arabidopsis*) and both structural and biochemical approaches are needed to fully characterize these transporters and evaluate the importance of glycosyl groups for substrate specificity. For example, the transporters characterized by Miao and Liu (2010) were located in *Arabidopsis* rosette leaves that contain only small amounts of lignins, and were shown to be mainly associated with the transport of *p*-coumaryl alcohol, a minor lignin component. More recently, Tsuyama et al. (2013) showed that a light membrane fraction prepared from differentiating xylem of hybrid and wild poplar, Japanese cypress and pine had clear ATP-dependent transport activity specific for coniferin suggesting the existence of a common transport mechanism dependent on a proton gradient created by a vacuolar-type H^+^ ATPase (V-ATPase) in the lignifying tissues of both woody angiosperms and gymnosperms. Considering the fact that these V-ATPases were also located in the trans-Golgi network, it could be argued that coniferin may, in fact, be transported through the cell in small vesicles as suggested nearly 50 years ago by Pickett-Heaps (1968). It still remains necessary to determine whether the transport systems are universal or are dependent on molecules and/or species.

When considering the compartmentation of phenylpropanoids within the plant cell, the location of the enzymes implicated in their glycosylation status should also be taken into account. It seems clear that the absence of predicted signal peptides in plant UGT sequences supports the belief that they are located in the cytoplasm (Ross et al., 2001). The transfer of the glycosyl group on acceptor molecules can then be considered as exclusive to this compartment. On the other hand, the removal of sugar moieties by ß-glucosidase can potentially occur in a different compartment. Historically, the first ß-glucosidase capable of hydrolysing phenylpropanoid glycoconjugates was found in the cell wall fractions of roots and hypocotyls of *Picea abies* seedlings (Marcinowski and Grisebach, 1978) and cell suspensions of *Cicer arietinum* (Hösel et al., 1978). Since then, different ß-glucosidases have been identified in many plant species—generally by–omics approaches—but very few have been characterized at the biochemical level. It has been assumed for almost 40 years that the enzyme and the glycosylated substrate can be present in different locations and that contact between them occurs when the tissue or cells are damaged compromising cell integrity. This physical separation exists at the cellular level in sorghum seedlings where the cyanogenic glycoside dhurrin and a specific ß-glucosidase are located in different cells, the substrate in the epidermis and the enzyme in the mesophyll tissues (Kojima et al., 1979). However, it should be noted that due to the difficulty of physically separating these tissues, the results were obtained using protoplasts prepared from leaves and may therefore not represent the real situation *in planta*. In the case of phenylpropanoids, this separation generally occurs at the subcellular level. For example in *Melilota alba* leaves, coumarin (glycosylated form of 2-hydroxycinnamic acid) is located in the vacuole (also prepared from protoplasts) whereas the specific ß-glucosidase is found in the cell wall (Oba et al., 1981). The optimum pH determined for purified or recombinant ß-glucosidases exhibiting coniferin specificity in gymnosperms, monocots and dicots (Hösel et al., 1978; Marcinowski and Grisebach, 1978; Escamilla-Trevino et al., 2006; Baiya et al., 2014) generally ranges between 4.5 and 5.5 which is consistent with the acidic environment of the cell wall. The role of ß-glucosidases in cell wall lignification and plant defense responses is discussed further in the following section of this paper. The subcellular seperation is a critical need for molecules that are toxic under their aglycone form but may not be necessarily widespread. In rice, a ß-glucosidase showing *in vitro* activity against a *p*-coumaryl alcohol glucoside but not coniferin with an optimum pH of 6.5 was identified in the cytoplasm (Rouyi et al., 2014) suggesting that both glucosylation (by cytosolic UGTs) and deglucosylation (by cytosolic ß-glucosidases) may occur within the same cellular compartment (but not necessarily the same cell) at least in this species. Transglucosidases are GH1 enzymes that can transfer a sugar from a donor other than a nucleotide phosphate or phospholipid phosphate to an acceptor. Among these, the rice Os9BGlu31 enzyme is located in the vacuole of senescing flag leaves and developing seeds and is induced in seedlings in response to drought stress and different phytohormones (Luang et al., 2013). Subsequent functional characterization indicated that the enzyme showed the highest activity with an optimum pH of 4.5 toward 1-*O*-feruloyl-ß-D-glucose, as well as toward flavonoid glucosides but with lower efficiency. The presence of such an activity inside the vacuole would suggest that transported glycosylated phenylpropanoids can undergo further enzymatic modifications and so should not be considered as “dead-end” storage products. The detection of six GH1 glucosidases in *Arabidopsis* leaf vacuoles by proteomics also supports this idea (Carter et al., 2004).

In conclusion, a thorough understanding of the biological roles of (glycosylated) phenylpropanoids not only depends on detailed knowledge about their biosynthesis, but also on a better comprehension of their localisation and transport within, and between different tissues, cells and compartments.

## At the crossroads of monolignol glycosylation and lignin biosynthesis

There are still many uncertainties about whether pooled glucoconjugated monolignols—and by extension hydroxycinnamic acids and aldehydes—really constitute a stock for subsequent lignin construction. Terashima and Fukushima (1988) followed the incorporation of radiolabeled glycosylated monolignols in *Pinus thumbergii* by microautoradiography in softwood differentiating xylem. When incubated at concentrations of up to 1 mg glucoside in 100 μl, the labeled monolignols were incorporated in the cell wall leading the authors to conclude that glycosylated forms could contribute to the lignification process. Although, it is tempting to think that incorporation occurs after a simple hydrolysis of the sugar moieties, an alternative pathway involving the oxidization of coniferin to coniferaldehyde glucoside prior to the deglucosylation step, followed by the transformation to coniferyl alcohol was suggested by the use of radiolabeled coniferin in *Gingko biloba* shoots (Tsuji et al., 2005). Either way, these experiments show that exogenous coniferin can serve as a lignin precursor.

Lignin composition is modified when plants are submitted to a mechanical stress. Compression wood (CW) is formed on the lower part of stems and branches of inclined softwoods (Timell, 1986). When compared to normal wood (NW), CW tracheids contain less cellulose but more lignin with (in addition to G-units) a higher proportion of *p*-hydroxyphenyl units (H-units) that are almost absent in NW and opposite woods (Terashima and Fukushima, 1988; Donaldson, 2001). In addition to increased lignin content, Yoshinaga et al. (2015) also reported that coniferin was less abundant in compression wood than in normal wood and that the *p*-coumaryl alcohol glucoside was undetectable in the two Japanese softwoods *Chamaecyparis obtusa* and *Cryptomeria japonica*. In the light of these results the authors suggested that if glycosylated monolignols do indeed function as lignin precursors, then their incorporation as G- and H- subunits must occur immediately after their synthesis without being pooled in the cell. A possible relationship between the accumulation of glycoconjugated phenylpropanoids and lignin status has also been reported in flax. In this species, bast fibers located in the outer part of the stem possess very thick secondary cell walls containing low amounts (4% cell wall residue) of lignin compared to inner stem xylem cell walls containing high amounts of lignin (25% cell wall residue; Day et al., 2005). An ultra-high-performance liquid chromatography-Fourier transform ion cyclotron resonance-mass spectrometry (UHPLC-FT-ICR-MS) phenolic profiling approach showed that flax inner- and outer-stem tissues had different phenolic profiles (Huis et al., 2012). In total 81 different phenolic compounds including monolignols, oligolignols and (neo)lignans were identified and among these, 39 were glycosylated and present (except for one compound) in much higher amounts (up to 92-fold higher) in the outer stem tissues. Whether the observed differences in glycosylated phenolics content can be related to the differences in cell wall lignin content in the two stem tissues remains an interesting question for future research.

It is now well known that transgenic approaches targeting general phenylpropanoid or more specific monolignol genes can modify both lignin composition and quantity (Chabannes et al., 2001; Voelker et al., 2010). For example, inactivation of a caffeic acid *O*-methyltransferase (*COMT*) gene in *Panicum virgatum* decreased lignin content concomitantly with metabolites related to trans-sinapyl alcohol including the conjugation-product syringin (Fu et al., 2011; Tschaplinski et al., 2012). In *Arabidopsis*, a global view of the metabolic shifts in the lignin-related biosynthesis was obtained by a systems biology approach (Vanholme et al., 2012). These published data revealed that mutations in phenylpropanoid genes have different impacts on glycosylated monolignol production. In the *pal1, c4h, 4cl, ccoaomt1*, and *ccr1* mutants, the amounts of syringin and coniferin were significantly lower when compared to the wild type. All these mutants except for *pal1* also show reduced lignification. An increase in the syringin and coniferin levels was also reported in the *4cl2* and *f5h1* mutants respectively without any change in lignin contents. The overall effect depends on the mutated gene and the phenylpropanoid considered. A rather general tendency shows that the glycosylated metabolites beyond coniferaldehyde in the biosynthetic pathway tend to decrease in *pal1, c4h, 4cl1, ccoaomt1*, and *ccr1* and that the glycosylated metabolites produced before the mutated gene increase in *4cl1, ccoaomt1, ccr1*, and *comt*. The results obtained in this species clearly support the hypothesis that monolignols whose destiny is to become either glycosylated or polymerized as lignins are produced in a common metabolic pathway.

Comparison of lignin biosynthetic and UGT gene expression patterns reveals interesting co-expressions hinting at links between lignification and monolignol glycosylation. In Figure 3, the group 5 shows that two (*UGT72E1* and *UGT72E2*) of the three monolignol-associated UGT72E subfamily genes are coexpressed with the peroxidase *PRX49* and that all three members are coexpressed with *PRX72*. These two peroxidases were recently described as major actors of lignin polymerization in the cell wall (Herrero et al., 2013; Fernandez-Perez et al., 2015) and since both peroxidases and UGTs use the same substrates, the observed coexpression would strongly suggest the existence of a functional link between these enzymes. Further evidence for such an idea is provided by the effects of mutations in the triple mutant of 3 laccase genes (*LAC4, LAC11*, and *LAC17*) in *Arabidopsis* (Zhao et al., 2013). The growth of these plants is blocked in the early stages of development with (among other) phenotypic characters, a narrow root diameter and vascular development arrest. The authors attribute these severe growth perturbations to the lack of lignin and secondary cell walls in the mutants and to an overaccumulation of free forms of monolignols. Indeed levels of coniferin and syringin were both increased in the mutant along with those of sinapoylglucose and three kaempferol glycosides. Interestingly, the expression of most monolignol biosynthesis genes was not affected showing the lack of a negative feedback regulation on the transcription. On the other hand, the accumulation of monolignol glucosides in this triple mutant could be correlated to significant up-regulation of *UGT72E2* and *UGT72E3* potentially related to monolignol detoxification mechanisms in the absence of lignin polymerization.

The inversely proportional expression of *UGTs* and genes involved in lignin polymerization was also demonstrated in the *lbf1* flax mutant isolated from an ethyl methanesulfonate (EMS) population (Chantreau et al., 2014). In these plants, the bast fibers located in the external parts of the stems show ectopic lignification of their secondary cell walls. This additional production of lignin is accompanied by a higher *cinnamoyl-CoA reductase* (*CCR*), *caffeic acid O-methyltransferase* (*COMT*), *cinnamyl alcohol dehydrogenase* (*CAD*), and *PRX71* ortholog transcript accumulation indicating a possible increase in monolignol production and oxidation. In parallel, the reduction in the abundance of transcripts corresponding to a *UGT72E* ortholog observed in the outer tissues was explained by the reduced necessity for detoxification due to a higher incorporation of monolignols into the lignin polymer.

Altogether, these examples illustrate the fact that variations in lignin amounts can have a potential effect on monolignol glucoside production but the opposite is not necessarily true. The overexpression of *UGT72E2* and *UGT72E3* in *Arabidopsis* (Lanot et al., 2006, 2008) mostly led to increased accumulation of coniferin in light-grown roots but had little impact on syringin levels. Functional validation of both genes also came from the significant reduction in coniferin and syringin in down-regulated transgenic plants. Perhaps the most interesting result coming from these transgenic approaches is that regardless of the effects on monolignol glucoside levels, no impact on lignin was ever observed, even when the silencing of the three *UGT* genes by an RNAi approach led to a 90% reduction (Wang et al., 2013). These results strongly suggest that changes in monolignol glucosides do not affect lignin levels. Nevertheless, it is interesting to note that the heterologous overexpression of a poplar UGT gene in tobacco (Wang et al., 2012) led to an increase of lignin in stems. However, in this case the authors indicate that the corresponding recombinant enzyme was unable to glycosylate phenylpropanoid components suggesting that the observed effect was indirect.

Overexpression of *UGT72E2* and *UGT72E3* also led to the accumulation of coniferin and syringin in the leaves associated with a significant reduction in the amounts of sinapoyl malate. A link between monolignol glucosides and hydroxycinnamic acid esters was also suggested by the results obtained by Hemm et al. (2004) showing that the light-induced production of coniferin and syringin in roots is accompanied by up-regulation of the *4CL3* (At1g65060) gene involved in the production of sinapate esters (Nair et al., 2004). Interestingly, the analysis of the gene expression heatmap shown in Figure 3 (group 1) indicates that *4CL3* is coexpressed with the two glycosyltransferases *UGT84A1* and *UGT84A2* implicated in the production of sinapoyl glucose (Milkowski et al., 2000).

**Figure 4:**
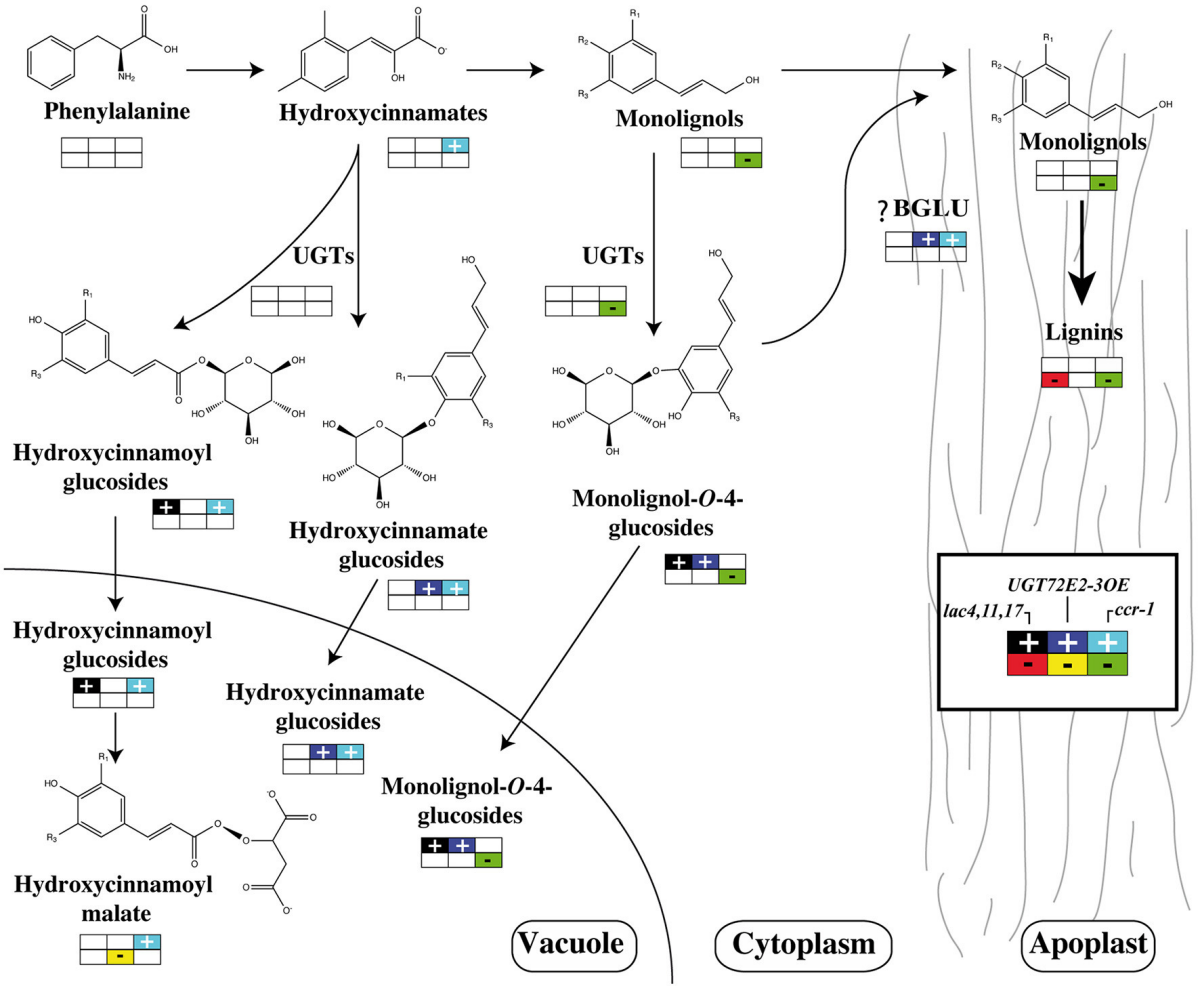
**Effects of phenylpropanoid gene mutations or UGT gene overexpression**. A schematic view of lignin, glucoconjugated monolignols, hydroxycinnamate glucosides, or hydroxycinnamoyl esters are presented in *ccr-1* and *lac4,11,17* mutants and *35S:UGT72E2-3* overexpressors. For metabolites and transcripts, a color code indicates an accumulation or a reduction compared to the wild-type. Data were extracted from Vanholme et al. (2012), Zhao et al. (2013), and Lanot et al. (2006, 2008) respectively.

As stated above any link between lignin and phenylpropanoid glucoconjugates should not only take into account UGT activities and gene expression but also ß-glucosidase behavior. In the systems biology approach developed by Vanholme et al. (2012), the decrease in the amounts of coniferin and syringin were nicely related to the lower *UGT72E2* and *UGT72E3* expression levels, but also to the increase in both *BGLU45* and *BGLU46* transcript accumulation. These two genes were shown to be specific for monolignol glycosides (Escamilla-Trevino et al., 2006) and the corresponding knockout mutants displayed a significant increase in stem coniferin content but, as for UGT overexpressors, no changes in lignin content were observed (Chapelle et al., 2012). A general overview of the effects of mutations of genes involved in monolignol production, polymerization or glycosylation is shown in Figure 4.

In conclusion, although monolignol glucosides have long been considered as participants in the lignification process (Freudenberg and Harkin, 1963; Freudenberg and Torres-Serres, 1967) it seems unlikely that the plant actually uses these molecules stocked in the vacuole to synthesize lignin. In contrast, the perturbation of genes involved in lignification has a clear impact on the production of glycoconjugated monolignols. However, it would be important to check whether the results obtained in *Arabidopsis* are also valid for woody plants. Although, secondary cell wall formation and especially lignin formation have been extensively studied in poplar, few studies (Wang et al., 2012) have addressed the potential links between phenylpropanoid glycosylation and lignification in trees.

## The role of glycosylation in phenylpropanoid function

### Glycosylation and phenylpropanoid stability

Many phenylpropanoids are toxic and unstable molecules and so rarely accumulate in their aglycone form in plant cells (Whetten and Sederoff, 1995; Alejandro et al., 2012; Väisänen et al., 2015). The mechanism of their toxicity is not clear but it has been suggested by Väisänen et al. (2015) that: (i) high concentrations of coniferyl alcohol could react with lipids or proteins in cellular environments in the presence of reactive oxygen species, (ii) since coniferyl alcohol could be easily degraded (e.g. to vanillin or ferulic acid) it is possible that its toxicity results from the accumulation of degradation products (iii) the availability of coniferyl-alcohol leads to the formation of a lignin-like compound and reduced growth of plant cells. More generally, aglycone forms of phenylpropanoids are nucleophilic molecules with high damage potentialities on diverse cellular components. The attachment of carbohydrate moieties will then reduce their reactivity and improve their stability (Jones and Vogt, 2001). In the case of monolignols, it is known that the conversion into their glycone forms by UGT activity reduces their toxicity (Bock, 2016). This is also the case of anthocyanidins for example, which only accumulate in cells in their glycosylated forms (Boss et al., 1996).

Glycosylation may not only prevent the toxicity of aglycone phenylpropanoids but can also contribute to the production of protectant molecules against reactive oxygen species (ROS). Flax seed coats contain the lignan secoisolariciresinol diglucoside (SDG) whereas their aglycone precursors pinoresinol, lariciresinol, and secoisolariciresinol are undetectable whatever the development stage considered (Hano et al., 2006). SDG was previously shown to prevent lipid peroxidation in a concentration-dependent manner (Prasad, 1997) and it is believed that it may play a similar role in the plant. This hypothesis is consistent with the concomitant presence of very high amounts of SDG and highly reactive polyunsaturated fats in the flax seeds (Lorenc-Kukula et al., 2005). Nevertheless, a peroxyl radical scavenging capacity comparable to that of glutathione has been described for the aglycone form of the isoflavone daidzein and the non-glycosylated lignan honokiol (Kim et al., 2010) suggesting that glycosylation is not essential for ROS protection capacity. Glycosylation can therefore contribute to reducing phenylpropanoid toxicity and increasing stability thereby explaining the widespread occurence of phenylpropanoids in plant development and resistance/tolerance to major biotic and abiotic stresses (Lim and Bowles, 2004; Bowles et al., 2005).

### Phenylpropanoid glycosylation and plant defense

The phenylpropanoid biosynthetic pathway is stimulated during the hypersensitive response (HR) induced by pathogen infection and is associated with the production of phenylpropanoid-derived phytoalexins such as coumarins and isoflavonoids (Hammond-Kosack and Jones, 1996; Dorey et al., 1997; Langlois-Meurinne et al., 2005). Chong et al. (2002) reported that the glycosylation of phenylpropanoids in tobacco played a significant role during Tobacco Mosaic Virus (TMV) infection and that the down-regulation of a tobacco glycosyltransferase gene (*TOGT1*) led to reduced TMV resistance. The authors suggested that scopolin (a glycoconjugated coumarin) is a storage form of the aglycone form scopoletin that functions as an antiviral agent potentially involved in scavenging ROS accumulated during the infection. Overexpression of the *TOGT* gene also enhanced resistance against the potato virus Y (PVY) in tobacco (Matros and Mock, 2004). Recent work also shows that sinapate esters, coniferin and the glycosylated pinoresinol and lariciresinol lignans accumulate when *Arabidopsis* leaves are infected by the soil-borne ascomycota *Verticillium longisporum* (König et al., 2014). When tested on agar plates, only the sinapoyl glucose and coniferyl alcohol, but not sinapyl alcohol, managed to inhibit the growth of the fungi, whereas coniferin specifically inhibited melanisation. The authors proposed that the growth-inhibiting differences observed between the two aglycone monolignols were solely due to the oxidization of coniferyl alcohol to ferulic acid, which was also shown to have an inhibitory effect on *V. longisporum*. Coniferin-accumulating transgenic plants over-expressing *UGT72E2* were less susceptible to the fungi. In this case, it was suggested that coniferin would operate as a storage form of coniferyl alcohol, which could further be transformed into ferulic acid. Plant species can also display glycosylation-related cultivar- and pathogen-dependent responses to biotic stresses. In wheat, genes encoding cytochrome P450 (CYP709C1) and a UGT were more strongly upregulated during the infection by *Fusarium graminearum* compared to *Magnaporthe grisea* and cultivar resistance to *F. graminearum* led to a stronger expression in incompatible (resistant) interactions with the chinese spring wheat cultivar Sumai 3 (Ha et al., 2016). Based on these observations the authors propose the existence of different resistance genes related to both fungi and confirm the necessity to combine genes in breeding programs to obtain multi-resistance plants as it was proposed by Schäfer et al. (1963). Deoxynivalenol produced by *Fusarium* is glycosylated by DOGT1 (UGT73C5) and the constitutive overexpression of the corresponding gene in *Arabidopsis* led to enhanced tolerance against this mycotoxin (Poppenberger et al., 2003). Other studies have underlined the importance of some UGT genes implicated in pathogen response and redox status during the pathogen infection. For example, it was reported that UGT73B3 and UGT73B5 are involved in the hypersensitive response to the bacteria *Pseudomonas syringae* pv tomato in *Arabidopsis* and participate in the regulation of the redox status of plant cells. However, the exact role of these UGT *in planta* remains unknown (Langlois-Meurinne et al., 2005; Simon et al., 2014).

There is now clear evidence that UGTs play an important role in disease resistance even though their precise contribution still remains unclear. However, it appears that the addition of a glycosyl group does not necessarily play a major role in determining the biological activity of phenylpropanoids and it may even block the biological activity of a particular molecule toward the pathogen. In this case, it is possible that glycosylation may play a role in maintaining the homeostasis of an ensemble of pathogen-specific molecules. Langenbach et al. (2013) suggested that disruption of the *UGT84A2/BRT1* gene can lead to an enhanced accumulation of certain sinapate derivatives or new metabolites absent from wild-type plants, which would confirm the important role of UGTs in maintaining a specific pool of phenylpropanoid-related metabolites.

### Phenylpropanoid glycosylation and abiotic stress

The *Arabidopsis* UGT84A2 and its *Brassica napus* ortholog UGT84A9 are both involved in the production of sinapoyl malate. This HCA glucose ester accumulates in vacuoles of sub-epidermal tissues and is believed to play a role in the protection against the harmful effects of UV-B radiation (Landry et al., 1995; Meißner et al., 2008; Dean et al., 2014). Li et al. (2010) isolated the *Arabidopsis* Pna-10 accession containing a 13-kb deletion that eliminates the *SNG1* gene responsible for the conversion of sinapoylglucose to sinapoylmalate, and the gene encoding sinapoylglucose:anthocyanin sinapoyltransferase (*SAT*). As a result, Pna-10 is unable to accumulate both sinapoylmalate and sinapoylated anthocyanins. Under field conditions, this mutation is not lethal suggesting that the presence of sinapoylmalate in the leaves may not be absolutely essential for plant protection. More recently, Hectors et al. (2014) highlighted the role of the rhamnosylated kaempferol and quercetin glycosides during UV acclimation in *Arabidopsis*. The concentration of these compounds increased as a result of UV-stress (and related oxidative stress) and could be correlated with the upregulation of the flavonol-7-O-rhamnosyltransferase *UGT89C1*. The biological role of these flavonoid glycosides is not clear as they are less effective anti-oxidants that the corresponding aglycone forms (Vogt and Jones, 2000; Gachon et al., 2005). The authors suggest that the accumulation of flavonol glycosides constitutes a reserve of flavonols which could be easily mobilized at any given time and especially under UV stress conditions.

A relationship between aglycone/glycone flavonoids and oxidative stress in *Arabidopsis* was also shown by Kim et al. (2010). In this case, the loss of function of three genes *UGT73B1, UGT73B2*, and *UGT73B3* involved in the glycosylation of flavonoids, led to a greater tolerance to oxidative stress whereas overexpression of *UGT73B2* increased the sensitivity to ROS. The significant role of HCA glycosylation was also highlighted in senescent and water stressed plants (Fini et al., 2012; Torras-Claveria et al., 2012). An accumulation of the coumarin esculetin and quercetin 3-O-glucosides was observed in water stressed *Fraxinus ornus* leaves simultaneously with a decrease in antioxidant enzyme activities (Fini et al., 2012). The authors suggested that these glycosides could act as H_2_O_2_ scavengers during water stress. These results are also consistent with those of Kylli et al. (2008) who showed that sinapic and ferulic acid glycoside esters were efficient antioxidants. More recently, Babst et al. (2014) showed an up-regulation of *UGT84A17* expression under environmental stress (in particular under N limitation) in transgenic *Populus*, leading to the accumulation of hydroxycinnamate glucose esters (especially caffeoyl-4-coumaroyl- and cinnamoyl-glucose esters). This accumulation was to the detriment of flavonoid glucoside synthesis, which also requires hydroxycinnamates in their free form as intermediates. These authors highlighted a metabolic trade-off associated with stress dependent phenylpropanoid glycosylation (Dauwe et al., 2007). Finally, a novel *Arabidopsis* glycosyltransferase gene *UGT85A5* (Sun et al., 2013) and more recently *UGT85U1* (Ahrazem et al., 2015) are both significantly induced by salt stress. Nevertheless, their connection with the phenylpropanoid pathway and their substrate affinity require more detailed characterization.

Although, the antioxidant proprieties of phenolic molecules have been known for a long time, the precise role(s) of glycosylation and, by extension the role of UGTs and ß-glucosidases in abiotic stresses is difficult to understand because of the existence of numerous signal pathways that can positively or negatively interact with each other. Such cross-talk is sometimes illustrated by the production of common glycosylated phenylpropanoids under different stresses. For example roots of hydroponically-grown *Arabidopsis* plants produce the glycosylated coumarin scopolin and monolignol coniferin when they are submitted to oxidative stress, root wounding, or nitrate deprivation (Ward et al., 2011). A major challenge is therefore to deepen our knowledge on the response mechanisms of plants to environmental changes in order to understand how these glycoconjugates can contribute to plant protection.

### Phenylpropanoid glycosylation: aroma, taste, and color of plant products

Flavonoid glycosylation plays a significant role in determining flower, leaf, seed, and fruit color necessary for attracting animals for flower pollination and/or seed dispersion (Tanaka et al., 2008). For example, strawberry fruits contain anthocyanins (glycosides of anthocyanidins) that give them the attractive red color in the ripe fruit (Griesser et al., 2008). Six anthocyanidins are generally present and their glycosylation is usually driven by UDP-glucose:flavonoid-O-glycosyl-transferase (UFGT) (Jaakola, 2013). However, the mechanism is quite complex and takes place in a species-specific manner. These anthocyanidins are most frequently O-glycosylated at the C3- followed by the C5-and/or C7-position (Tanaka et al., 2008). The glycosyl moieties of anthocyanins are themselves usually later modified by aromatic and/or aliphatic acyl moities. After these modifications, anthocyanins become stable compounds and accumulate in vacuoles. The range of these different mechanisms causes a variety of flower and fruit colors (Vogt and Jones, 2000; Grotewold, 2006; He et al., 2015; Li et al., 2016). In the case of strawberry fruits, Griesser et al. (2008) demonstrated that FaGT1, an anthocyanidin-3-O-glucosyltransferase, is a key enzyme in the phenylpropanoid biosynthesis because it channels the flavonoid pathway into anthocyanins and not into the glycosylated flavonols. Anthocyanidin 3-O-glucosyltransferases have also been characterized in many ornamental commercial plants (Tanaka et al., 1996; Yamazaki et al., 2002; Bowles et al., 2005; Sasaki and Nakayama, 2015). Recently, Matsuba et al. (2010) have shown that a novel glucosylation reaction on anthocyanins occurs with 1-*O*-β-D-vanilly-glucose as the sugar donor molecule in the petals of carnation and *Delphinium*.

The glycosylation of flavonoids may also be responsible for fruit flavor and aroma (Frydman et al., 2013; Tikunov et al., 2013; Hjelmeland and Ebeler, 2015). In Citrus, bitter species (e.g. pummelo, grapefruit) accumulate bitter flavonone 7-O-neohesperidosides (neohesperidin and naringin) which are synthesized through 1,2 rhamnosyltransferase activity and non-bitter species (e.g. oranges, mandarins) accumulate flavanone-7-O-rutinosides (hesperidin and narirutin) synthesized by 1,6 rhamnosyltransferases (Frydman et al., 2013). The roles of these different glycosylated flavonoids have not yet been determined but Del Rio et al. (2004) suggested that they are involved in plant defense against pathogens. Phenylpropanoid volatiles or volatile organic compounds (VOCs) are responsible for fruit aroma (Hjelmeland and Ebeler, 2015). In the case of green tomatoes, the aroma is termed “smoky” (Tikunov et al., 2013) and results from the presence of volatile phenylpropanoid diglycosides (with a hexose pentose sugar moiety), which can be easily cleaved. In mature fruits, glycosylation is more complex and triglycosides are synthetized (with a dihexose-pentose moiety) that are less easily cleaved. Tikunov et al. (2013) have characterized a glycosyltransferase named NON-SMOKY GLYCOSYLTRANSFERASE1 (NSGT1) in ripe fruits unable to produce and release the “smoky” aroma. NSGT1 is unable to glycosylate the aglycone form of phenylpropanoids and is only involved in further elongation of the glycosidic moiety of glycosides. The physiological and ecological roles of these aromas have not yet been elucidated. Some authors propose that they may repel (in the case of green fruits) or attract (in ripe fruits) animals (Borges et al., 2008). Glycosyltransferase activities would therefore indirectly affect seed dispersion.

## Conclusions

In response to a wide range of developmental and environmental cues, plants continuously produce (and modify) an extremely large pool of different phenylpropanoid-based secondary metabolites thereby contributing to the success of these autotrophic and, in many cases, sessile organisms. Many plant phenylpropanoids are also of economic interest for humans and a better understanding of the different factors controlling their biosynthesis and compartmentation should facilitate production and plant improvement. It is now becoming clear that the synthesis, localisation and biological activity of plant phenylpropanoids is not only regulated via the transcriptional and post-transcriptional regulation of different biosynthetic genes/enzymes, but also involves the reversible glycosylation of these molecules by regioselective glycosyltransferases (GTs) and glycosyl hydrolases (GHs). A major future research challenge will be to obtain a better understanding of how plants regulate the individual members of these multigenic GT/GH families during development and in response to abiotic and biotic stress. Given the wide diversity of phenylpropanoid structures across the plant kingdom it will be important to obtain information on a number of different species. In addition it will also be necessary to explore the links between GT/GH regulation and the regulation of other genes/enzymes involved in determining phenylpropanoid availability and activity (e.g. phenylpropanoid biosynthesis, peroxidases/laccases, transporters, UDP-sugar production). Such knowledge will be particularly valuable for maintaining/improving cultivated plant production in a context of increased atmospheric CO_2_ levels, global warming and modified pathogen biodiversity.

## Author contributions

All authors planned the manuscript contents, collected data from the literature and drafted the manuscript. GN and JL produced the figures. GN and SH finalized the first submitted version and the revised manuscript.

## Funding

We are indebted to the Research Federation FRABio (Univ. Lille, CNRS, FR 3688, FRABio, Biochimie Structurale et Fonctionnelle des Assemblages Biomoléculaires) for providing the scientific and technical environment conducive to achieving this work. JL was financed by the French Ministère de l'Education Nationale, de l'Enseignement Supérieur et de la Recherche.

### Conflict of interest statement

The authors declare that the research was conducted in the absence of any commercial or financial relationships that could be construed as a potential conflict of interest.

## Supplementary material

Supplementary Figure 1 **Co-expression of UGT and phenylpropanoid genes**. The heat map was constructed using ATTED-II (Obayashi et al., 2009). When the mutual rank (MR) value <50, co-expression between the genes is considered as strong; if 51 <MR <1000, there is co-expression and if MR>1001, there is no co-expression.
