# Supplementary figures and images for: Glycosylation Is a Major Regulator of Phenylpropanoid Availability and Biological Activity in Plants
Source: Front Plant Sci. 2016 May 26;7:735. doi: 10.3389/fpls.2016.00735 (PMC4880792; doi:10.3389/fpls.2016.00735)

Data expression

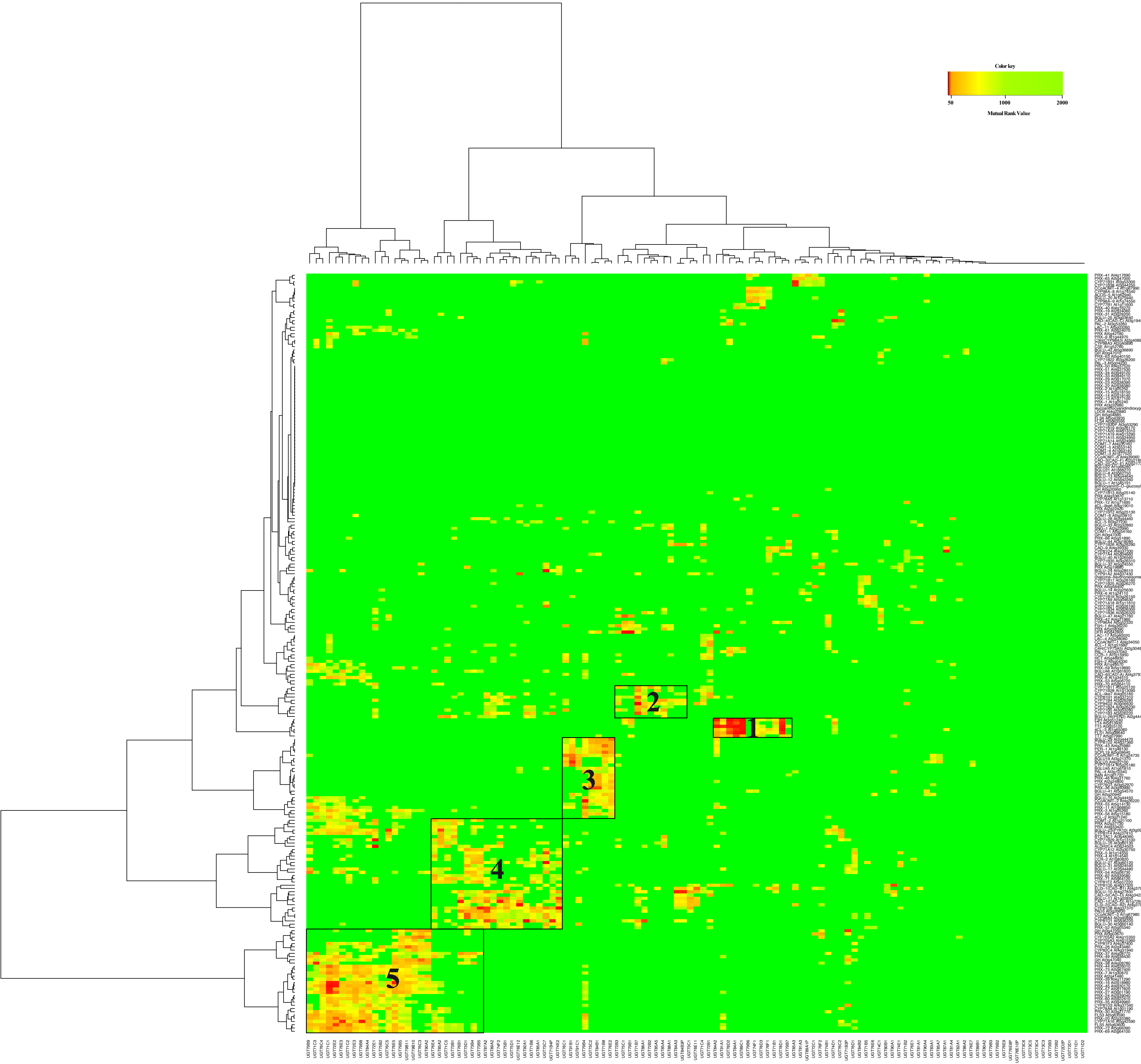

Supplement: Supplementary Figure 1 — Co-expression of UGT and phenylpropanoid genes. The heat map was constructed using ATTED-II (Obayashi et al., 2009). When the mutual rank (MR) value <50, co-expression between the genes is considered as strong; if 51 <MR <1000, there is co-expression and if MR>1001, there is no co-expression. [file Image1.PDF]
